# Old drugs for a new indication: a review of chloroquine and analogue in COVID-19 treatment

**DOI:** 10.1097/j.pbj.0000000000000132

**Published:** 2021-06-14

**Authors:** Teddy S. Ehianeta, Richard O. Akinyeye, Joshua I. Orege, Onome Ejeromedoghene, Adeniyi P. Adebule, Bright O. Okonkwo

**Affiliations:** a Institute of Biological Chemistry, Academia Sinica, 128, Academia Road Sec. 2, Nangang, Taipei 115, Taiwan; b Department of Industrial Chemistry, Ekiti State University, P.M.B. 5363, Ado-Ekiti, Ekiti State, Nigeria; c Dalian National Laboratory for Clean Energy, Dalian Institute of Chemical Physics, Chinese Academy of Sciences, Dalian 116023; d School of Chemistry and Chemical Engineering, Southeast University, Jiangning District, Nanjing, Jiangsu Province 211189; e CAS Key Laboratory of Nuclear Materials and Safety Assessment, Institute of Metal Research, Chinese Academy of Sciences, Shenyang 110016, China.

**Keywords:** chloroquine, clinical trials, COVID-19, drug repurposing, hydroxychloroquine

## Abstract

As an innovative therapeutic strategy, drug repurposing affords old, approved, and already established drugs a chance at new indications. In the wake of the COVID-19 pandemic and the accompanied urgency for a lasting treatment, drug repurposing has come in handy to stem the debilitating effects of the disease. Among other therapeutic options currently in clinical trials, chloroquine (CQ) and the hydroxylated analogue, hydroxychloroquine (HCQ) have been frontline therapeutic options in most formal and informal clinical settings with varying degrees of efficacy against this life-threatening disease. Their status in randomized clinical trials is related to the biochemical and pharmacological profiles as validated by in vitro, in vivo and case studies. With the aim to bear a balance for their use in the long run, this review not only synopsizes findings from recent studies on the degrees of efficacy and roles of CQ/HCQ as potential anti-COVID-19 agents but also highlights our perspectives for their consideration in rational drug repositioning and use.

## Introduction

**Figure 1:**
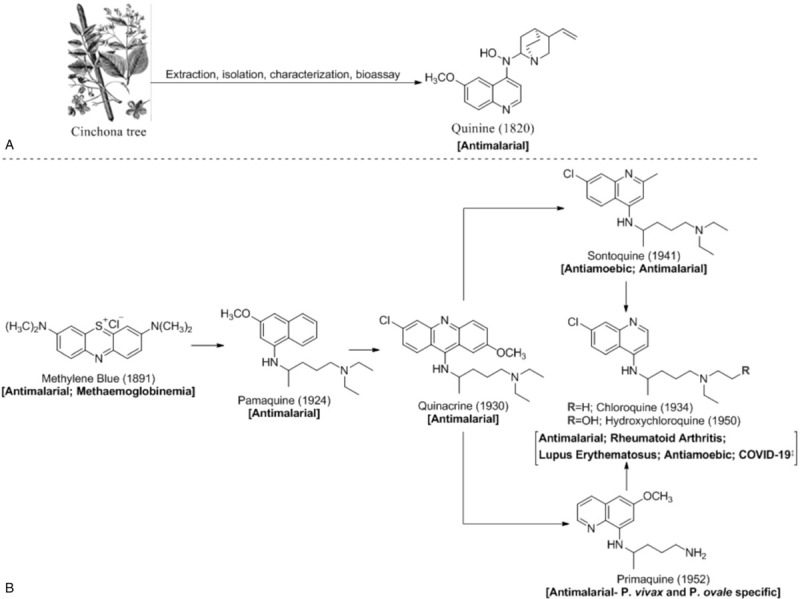
Schematic representation for the natural (A) and synthetic (B) development of chloroquine and hydroxychloroquine with the established indications and new indication^‡^

The beginning of the decade ushered in the outbreak of the novel Coronavirus Disease-2019 (COVID-19), a public health concern of global scale that transitioned quickly from an epidemic that originated in Hubei Province, Wuhan, China to a global pandemic within weeks. The disease caused by the Severe Acute Respiratory Syndrome Coronavirus type-2 (SARS-CoV-2),^1^ is associated with significant morbidity and mortality^2^ and there is currently no approved drug for treatment. While there are heightened research efforts and urgency to develop therapeutic options and vaccines that offer long term benefits, drug repurposing appears to be a veritable short-term approach. As an innovative strategy, drug repurposing (DR) affords old approved and already established drugs a chance at new indications, thus saving cost, time and resources that usually accompany the conventional drug development processes. In addition, repurposing drugs serves to cushion the mortality and Case Fatality Ratio (CFR) in the event of sudden outbreaks such as seen with COVID-19. In this regard, chloroquine (hereafter designated as CQ), a synthetic succedaneum of the cinchona alkaloid quinine (Fig. 1A) first isolated from the Cinchona tree bark has been a front-line drug in the fight against COVID-19. CQ is currently an 86-year old molecule first synthesized in Germany by Bayer as an effective substitute for natural quinine to treat malaria. Hydroxychloroquine (hereafter designated as HCQ), the 70-year old hydroxylated analogue of CQ have also been found to limit the total burden of COVID-19^3,4^ in 2020. In addition to its repositioning as treatment for autoimmune diseases, CQ has been reported to have a broad-spectrum of antiviral activities.^5,6^ Both therapeutic agents have developed through important precursors over the years beginning with Methylene blue once used as antimalarial (Fig. 1B). This paper reviews recent literature for the attendant pharmacological roles of CQ and HCQ in the management of the novel disease, general theories and guidelines for clinical trials and representative trials using CQ/HCQ as intervention agents since the outbreak of the disease. Furthermore, our expert views have been supplemented in some salient areas of the COVID-19 conversations.

## General biochemical and mechanistic profile of chloroquine and hydroxychloroquine

It is not unlikely that the multi-faceted mechanisms of CQ and HCQ easily positions these age-long molecules as repurposed drugs for COVID-19, acting at different stages of the host-virus cycle. For one, the profile of CQ and HCQ interfering with viral entry into host cells by inhibiting quinone reductase 2, a precursor for sialic acid biosynthesis^7^ is a step quite early in the mechanistic course. Indeed, sialic acid has been implicated in viral attachment and entry into host cells as seen with human coronavirus (hCoV-OC43) and Middle East Respiratory Syndrome Coronavirus (MERS-CoV).^8,9^ CQ is a blood schizonticidal drug without activity against the liver stage^10^ known to block virus infection by increasing endosomal and lysosomal pH required for viral fusion and replication,^11^ as well as interfering with the glycosylation of cellular receptors of SARS-CoV.^12,13^ Currently, there are conversations among glycochemists and glycobiologists on what sugars decorate the surface of the spike (S) proteins of the SARS-CoV-2 and their order of alignment. This discourse is now ahead of the initial conception of the role of glycans on the structural milieu of the virus. This structural concept is not only important in understanding the pathogenesis of the disease but also in elucidating more detailed mechanisms of *de novo* new molecular entities (NME) and repurposed drugs. It is now becoming common knowledge that the SARS-CoV-2 utilizes the glycan motif as a camouflage, thus by-passing the host immune system. It is likely that this glycan shield enhances the spread of virus within a network of uninfected cells within the host. By raising endosomal pH, thus inhibiting a pH-dependent virus-endosome fusion, alkaline CQ and HCQ can also inhibit early stage viral replication.^14^ It has also been established based on *in silico* studies, that the S protein of the SARS-CoV-2 not only uses the cellular receptor angiotensin converting enzyme 2 (ACE2) for viral entry but also uses the sialic acids and gangliosides. Indeed, CQ and HCQ bind to these glycans with great affinity, thus inhibiting glycosylation, viral fusion, entry and consequently replication.^15^ In a personal view Communication, Savarino and colleagues hypothesized that by CQ/HCQ inhibiting the production of proinflammatory cytokines that are implicated in COVID-19 infection, they mitigate the cascade of pathological events leading to Acute Respiratory Distress Syndrome (ARDS) early enough.^16^ Like CQ in other mechanistic profiles, HCQ in addition also inhibits major histocompatibility complex (MHC) class II expression which inhibits T cell activation expression of CD145 and cytokines release. So far, it is likely that the inhibition of the so-called cytokine storms is the rate limiting step in the progression of the disease from mild to severe, and also the focus of most drugs repurposed as potential anti-COVID-19 agents.

## Efficacy of chloroquine and hydroxychloroquine in COVID-19 studies

Chloroquine is enlisted in the model list of essential medicines of the World Health Organization (WHO) owing to its affordability and established clinical safety profile in the treatment of malaria and conditions for which it is indicated.^17^ However, the efficacy and safety of CQ and HCQ in the treatment of SARS-CoV-2 remains debatable despite *in vitro* studies that showed both agents to be effective against several viruses, including Severe Acute Respiratory Syndrome Coronavirus (SARS-CoV).^5^ HCQ is used in autoimmune diseases such as rheumatoid arthritis and lupus, with *in vitro* antiviral activity against SARS-CoV-2 (EC_50_ of 0.72 μM).^4^ Relative to Remdesivir, Xiao and co-workers showed that in a time-of-addition (TOA) experiment, chloroquine acted at both entry and post-entry stages when Vero E6 cells were pre-treated with the 2019-nCoV virus at a multiplicity of infection (MOI) of 0.05.^18^ This results and similar *in vitro* studies demonstrated the efficacy of CQ which informed its use in several clinical trials in different climes of the world. Their result also speculated that the immunomodulatory effects of CQ may have a role to play in the antiviral activity in *in vivo* assays. However, a recently published *Nature* article evaluated HCQ both *in vitro* and in SARS-CoV-2-infected macaques and submitted that HCQ displayed antiviral efficacy in VeroE6 (African green monkey kidney cells) but not in the virus-infected macaques.^19^ It is not unlikely that the efficacy of HCQ in this regard is dependent on the genetic disposition of the test subjects. In this context, the genotypic rather than phenotypic variations will be a key consideration en route human clinical studies.

Preliminary clinical data by Guatret et al. indicated that intake of HCQ 600 mg daily cured 70% of the patients (no = 20) after 1 week.^13^ Fan and co-workers advocated the use of CQ 100 mg daily or HCQ 300 mg weekly as a chemoprophylactic especially by frontline health workers exposed to COVID-19, with promising results and attendant low prevalence of side effects in long-term use.^20^ In another Chinese study, a few severe adverse effects were seen in more than 100 COVID-19 patients when treated with CQ phosphate and showed marked efficacy upon treatment by improving their clinical outcomes and shortening their hospital stay.^21^ On the premise of the *in vitro* potency of HCQ (EC_50_ = 0.72 μM) over CQ (EC_50_ = 5.47 μM),^4^ Raoult and colleagues designed an open-label, non-randomized clinical trial where they reported an absolute virological cure rate in patients treated with HCQ and azithromycin combination therapy (100%) relative to HCQ monotherapy (57.1%) and control group (12.5%) (*P* < .001).^13^ The group excluded patients with histories of G6PD deficiency, QT prolongation and retinopathy. An interesting feature of the penultimate study is the adoption of Physiologically Based Pharmacokinetic (PBPK) models from their in vitro results to extrapolate a rationale HCQ dose for COVID-19 patients. This is a highly recommended protocol for other trials and agents currently undergoing recruitement. Unlike CQ, HCQ has enjoyed more inclusion in combination therapies to address this public health menace as can be seen with Azithromycin and Ivermectin.

A unified view of experts^22^ on the phosphate of CQ's use in the treatment of COVID-19-mediated pneumonia made 5 key recommendations based on findings from clinical trials that: (i) provided that there are no contraindications to the drug, a dose of 500 mg of CQ phosphate tablet taken twice daily for 10 days should be administered to patients with varying degrees of SARS-CoV-2 pneumonia infection; (ii) precautions not limited to blood testing to rule out the development of anemia, thrombocytopenia or leukopenia as well as serum electrolyte disturbances and/or hepatic and renal function dysfunction; (iii) routine electrocardiography to exclude the development of prolonged QT interval or bradycardia (iv) clerking patients to establish visual and/or mental disorientation; (v) avoidance of concurrent administration of drugs implicated in QT interval prolongation, such as antiarrhythmic drugs,^23^ antipsychotic medications,^24^ tricyclic antidepressants,^24^ fluoroquinolones,^25^ iv pentamidine,^26,27^ azole-derived systemic antifungals^28^ and macrolide antibiotics.^29^

**Figure 2:**
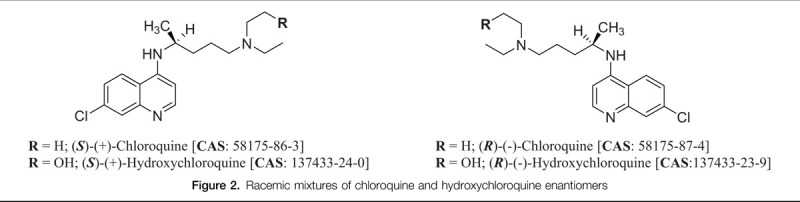
Racemic mixtures of chloroquine and hydroxychloroquine enantiomers

Generally, CQ and HCQ are chiral molecular entities administered as racemic mixtures, that is, each as a 1:1 mixture of 2 paired enantiomers, namely (*S*)-(+) and (*R*)-(–) (Fig. 2). As enantiomers, they can interact uniquely with receptors to elicit different clinical outcomes. McConathy and Owens communicated the significant effects of stereochemistry on the pharmacokinetic (Absorption, Distribution, Metabolism, Elimination and Toxicity- ADMET) and pharmacodynamic dispositions of a drug. They posited that although enantiomers of a chiral drug have identical physicochemical properties in an achiral environment, in a chiral environment however, one enantiomer is likely to demonstrate different pharmacological and chemical behavior than the other enantiomer.^30^

Since biological systems are in themselves chiral, each of the enantiomers of a chiral molecule can behave differently *in vivo*. Having established that the binding of CQ to plasma is enantioselective, Ofori-Adjei and co-workers further hypothesized that the binding efficiency of the (*S*)- relative to the (*R*)-enantiomer might be due to the preferential affinity of the (*S*)-enantiomer for plasma proteins such as lipoproteins and globulins.^31^ It is highly suggestive that the pharmacological profiles of stereospecific CQ and HCQ enantiomers will be plausible alternatives in the treatment of this disease without the cardiotoxic concerns that could serve as an exclusion criterion in COVID-19 clinical trials (vide infra). Similar to other racemates, it is presumptive that a crosstalk exists between a component of the racemic mixture and the undesirable effects of CQ and HCQ.

## Adverse effects of CQ and HCQ in the face of COVID-19

Following the WHO pronouncement of the pandemic status of COVID-19, there have been informal calls for accelerated large-scale production of CQ and HCQ along with accelerated small pool-size clinical trials. These calls were short-lived by the ethical concerns already associated with accelerated large-scale clinical trials of drugs with unproven efficacy.^32^ The heightened possibility of CQ-resistant strains of *Plasmodium falciparum* was also of concern in some quarters.

Typically, CQ and HCQ presents with relatively few adverse effects at clinical doses used for malaria. They however demonstrate serious side effects at high doses, when combined with other medicines or when administered parenterally. The prevalent adverse effects are eye-related (typically retinopathy and bilateral pigmentary retinopathy for CQ and HCQ respectively),^33,34^ cardiomyopathy,^35–37^ neuropathy,^38,39^ HCQ-induced myopathy,^40^ and Amodiaquine-mediated hypoglycaemia. A key manifestation of CQ and HCQ cardiotoxicity is atrioventricular (AV) block with prolongation of the QT(c) interval and short torsade de pointes.^41^ In their study titled, “Chloroquine-Induced QTc Prolongation in COVID-19 Patients”, Broek observed that CQ treatment for COVID-19 patients significantly prolonged the QTc interval by 34 to 35 ms after investigating a total of 95 patients with a baseline ECG recording and at least one ECG recording during CQ therapy. Their results showed that 23% of patients had a QTc interval exceeding 500ms while none of the patients had a prolonged QTc interval prior to the initiation of CQ therapy, thus validating the need for ECG monitoring before and after CQ administration.^42^ Similar to the curative intent, HCQ is often preferred to CQ due to its relative lower toxicity,^43^ and the latitude for large dosing and combination therapy. This preference was alluded to by our search of COVID-19 clinical trials (Table 1 vide infra) *on clinicaltrials.gov.*

**Table 1:** Completed and ongoing representative clinical trials using CQ and HCQ for the treatment of COVID-19

S/N	Clinical trial ID	Number of Enrolments	Interventions	Location	Method	Phase	Status	References
1.	NCT04325893	1300	Day1: HCQ 400 mg immediately after inclusion to study + 400 mg evening of day 1; Day 2-9: 200 mg 12 hourly	France	Double, randomized trial	Phase III	Ongoing (April 1–September 2020)	Database of U.S. National Library of Medicine (*ClinicalTrials.gov*) (accessed on 30 June 2020)
2.	NCT04328493	250	CQ phosphate based on body weights	Vietnam	Multi Center Randomized Open Label Trial	Phase II	Ongoing April 7, 2020–April 1, 2022	
3.	NCT04334148	15,000	HCQ 600 mg 12 hourly loading dose on day 1 followed by 400 mg on days 2–30	US	Double blind, placebo-controlled, randomized clinical trial.	Phase III	Ongoing (April 22–July 2020)	
4.	NCT04434144	116	HCQ 400 mg first day then 200 mg 12 hourly for 9 d + Azithromycin 500 mg daily for 5 d	Bangladesh	Prospective	Completed	May 2–June 5, 2020	
5.	NCT04342221	220	Day 1: HCQ Sulfate 800 mg. Day 2–7: 600 mg daily	Germany	Randomized Controlled Trial	Phase III	Ongoing (March 29, 2020–February 2022)	
6.	NCT04321278	440	(i) HCQ (ii) HCQ+ Azithromycin	Brazil	Randomized, Open trial	Phase III	Ongoing (March 28–August 30, 2020)	
7.	NCT04384380	45	Day 1: HCQ Sulfate 400 mg 12 hourly; Day 2–7: 200 mg 12 hourly	Taiwan	Multi-center, Randomized, Open label, Controlled Trial	Phase IV	Ongoing (April 1–September 30, 2020)	
8.	NCT04261517	30	HCQ 400 mg daily for 5 d	China	Randomized Open label trial	Phase III	Completed (February 6–February 25, 2020)	
9.	NCT04308668	1309	HCQ 800 mg orally once, followed by 600 mg after 8 h; then 600 mg daily for 4 d.	Canada	Pragmatic Randomized Clinical Trial	Completed	March 17–May 20, 2020	
10.	NCT04303507	40,000	Loading dose of CQ or HCQ: 10 mg base/kg/body weight randomized with placebo (1:1)	UK and Thailand	Double-blind, randomized, placebo-controlled trial	Ongoing	April 29, 2020–April, 2021	
11.	NCT04331600	400	CQ, in combination with telemedical approach	Poland	Multicenter, Randomized, Open label, Non-commercial	Phase IV	Ongoing (April 6–December 31, 2020)	
12.	NCT04344951	60	CQ phosphate 200 mg	Greece	Prospective, Open label	Phase II	Ongoing (April 6–April 30, 2021)	
13.	NCT04353336	40	CQ phosphate 200 mg	Egypt	Randomized, Open label	Phase II & III	Ongoing (May 23, 2020–December 1, 2030)	
14.	ChiCTR2000029939	50	CQ phosphate according to national guidelines	China	A Single-blind, Randomized, Controlled Clinical Trial	N/A	Ongoing (February 6, 2020–February 6, 2021)	
15.	ChiCTR2000029740	54	HCQ	China	Randomized, Open label controlled clinical trial	Phase IV	Completed (February 11–February 29, 2020)	Chinese Clinical Trial Registry (accessed on 1 July 20)

## Theories and guidelines governing clinical trials

Generally, safety evaluation and clinical safety of medical interventions are based on the measurement and analysis of previously reported clinical outcomes.^44^ Although the clinical safety of old drugs has been proven, some are not without adverse effects even when administered at clinical doses. For example, CQ and HCQ may present with cardiotoxicity such as arrhythmia, which can be life threatening. Hence, risk-benefit assessment should be key consideration with old drugs for new indications. An effective clinical trial should be designed to actively, cautiously and scientifically reflect the basic principles of the Helsinki Declaration and its regulations and laws.^45^ It is our submission that despite the urgency instigated by the wide-spread of the COVID-19, an ideal trialed drug is one characterized by rigorous evidence and strict adherence to the outlined guidelines for statistical clinical trials and the basic principles of randomization, control and replicable outcomes. In a systematic review, Cortegiani et al. posited that the clinical use of investigational drugs in COVID-19 patients should strictly be guided by the Monitored Emergency Use of Unregistered Interventions (MEURI) framework or after ethical approval as a trial as stated by the WHO.^3^ In the interim, the emergency authorization of CQ and HCQ, so far, by the FDA for their use does not indicate approval for mass usage in treating COVID-19. Therefore, treatment should be carefully considered, with the risks and benefits verified with proper monitoring of parameters.^46^ Based on a preliminary analysis of the data from the Randomized Evaluation of COVID-19 Therapy (RECOVERY) study, the COVID-19 treatment guidelines panel recommends against the use of CQ and/or HCQ for the treatment of COVID-19, except in a clinical trial. This might be connected to the unpredictable outcomes of CQ/HCQ effect on viral replication. A case in point is the promising in vitro antiviral activity of CQ displayed against the chikungunya virus (CHIKV)^47,48^ but enhanced α-virus replication in some animal models.^49,50^ This oddity is suggested to be due to the immunomodulatory and anti-inflammatory properties of CQ in vivo.^51^

## COVID-19 clinical trials using CQ and HCQ as intervention agents

A randomized clinical trials (RCT) is a comparative, quantitative study performed under controlled conditions accompanied by random allocation of interventions to comparison groups, thus making it the most rigorous and unbiased research method for determination of a cause–effect relationship between an intervention and an outcome.^52^ Since evidence from RCT remains the highest level of proof guiding impersonal practice decisions,^53^ clinical decisions therefore require evidence-based medicine to validate choices afterward.^54^ There has been avalanche of clinical trials since the outbreak of the disease, many of which have been archived in the *clinicaltrials.gov* and other authorized repositories. Our due search enlisted representative RCTs that employed CQ and HCQ as the intervention medicaments in COVID-19 test subjects (Table 1). As can be seen in Table 1, the highest and lowest enrolments are 40,000 and 30 respectively. It follows that clinical efficacies of CQ/HCQ which are often considered unreliable might be due to the underpowered enrollment typified in small groups. Conversely, large-numbered RCTs with well-founded efficacy profile for CQ/HCQ are likely to take longer time to complete recruitment, further prolonging the anticipated results. As earlier mentioned, owing to the peculiarity of the SARS-CoV-2, the pathogenicity of the disease and the varying efficacy profiles from *in vitro* studies, the genetic pool of the enrolments are likely confounders to the clinical outcomes and should be keenly considered in the design of repositioned agents and NMEs.

## Conclusion/perspectives

Our search indicated that overall, while more clinical data are available for CQ, majority of reported case series and trials administered HCQ even though both are therapeutically equivalent options for COVID-19. This preference alludes to the risk-benefit balance as a foremost consideration with old drugs for new indications. The suitability of CQ and HCQ as repurposed anti-COVID-19 agents has been a hugely debatable subject since the outbreak of the disease. However, in settings where these agents have been used, the risk-benefit margin is secondary to its efficacy in treating the disease. The authors hold the view that the varying efficacy profiles of HCQ against SARS-CoV-2 bench-marked by studies on Vero E6 cells (African descent) and Macaques (Oriental and Caucasian descent) suggests correlations between the therapeutic outcomes and genetic disposition of the test subjects, a pointer to pharmacogenomic approach to tackling disease. The pharmacological profile in view of the stereospecificity of CQ/HCQ is also an effective strategy to increase the safety-toxicity ratio. In addition, nano-dispersed forms of stereoselective enantiomers of CQ/HCQ with rationale combination therapies is an area yet to be exploited. There is still paucity of data on the pharmacokinetic assays of CQ and HCQ in COVID-19 patients for justifiable reasons. We also submit that despite the urgency instigated by the global wide-spread of this disease, an ideal trialed drug even if repurposed is one characterized by rigorous evidence and strict adherence to outlined guidelines for statistical clinical trials with the basic principles of randomization, control and replicable outcomes. Monitored administration of these agents can stem the tides of CQ/HCQ resistance in a post-COVID era. In effect, multi-disciplinary research is a go-to strategy to properly fashion out the complete efficacy of the use of CQ, HCQ and their analogues in COVID-19 management that will fully give backing to its strengths, weaknesses, opportunities and threats.

## Acknowledgements

We acknowledge the painstaking efforts of the anonymous reviewers toward the enhanced improvement of this review

## Authors’ contributions

TE conceptualized the idea; TE and ROA outlined the sections of the manuscript; TE, ROA, JIO and OE developed the different sections of the manuscript; APA and BOO developed the abstract, Introduction and Conclusion; TE, ROA, JIO, OE, APA and BOO critiqued the submissions from case series and clinical trials; TE and ROA reviewed and edited the article.

## Conflicts of interest

The authors declare that they have no competing interests.
